# Supplementary material for: PTP61F Mediates Cell Competition and Mitigates Tumorigenesis
Source: Int J Mol Sci. 2021 Nov 25;22(23):12732. doi: 10.3390/ijms222312732 (PMC8657627; doi:10.3390/ijms222312732)
Supplement: Supplementary file 1 [file ijms-22-12732-s001.zip › Supplementary Table S1 - fly stocks v3.pdf]

Supplementary Table S1. Fly stocks. This table contains the details of all the *Drosophila melanogaster* stocks and/or transgenic lines used in this study.

Designation	Alternate designations	Source	Stock IDs and Additional Information	References
hsFLP^{122} *; ; Ubi-GFP, FRT80B*		1620 was from Leonie Quinn	Constructed by combining BDSC stocks: *w*$^{-}$ *; Ubi-GFP, FRT80B* (1620) and *hsFLP*^{122}*, y*$^{-}$*, w*$^{-}$ *; vasa-phiC31* (23649)	
Ptp61F$^{\Delta}$		Coral Warr		Buszard et al., 2013
FRT80B		BDSC	2035	
FRT82B		BDSC	1988	
y^{-}*, w*$^{-}$*, eyFLP*^{2} *; Act>y*$^{+}$*>GAL4, UAS-GFP ; FRT82B, tub-GAL80*	*MARCM82B*	Original stock was from Xianjue Ma	Constructed by combining MARCM stock from Xianjue Ma with BDSC stock: *eyFLP*^{2}*, w*$^{-}$*, y*$^{-}$ (5580)	
y^{-}*, w*$^{-}$*, eyFLP*^{2} *; Act> y*$^{+}$*>GAL4, UAS-GFP ; FRT82B, tub-GAL80, scrib*^{1}	*revMARCM82B*	Original stock was from Xianjue Ma	Constructed by combining BDSC stock 5580 with stock from Xianjue Ma	
scrib^{1}		David Bilder		Bilder & Perrimon 2000
UAS-Ptp61F$^{isoB2.1}$	*Ptp61Fn*$^{2.1}$, *Ptp61Fn*	Coral Warr	Nuclear-localised isoform, 2nd chromosome insertion (2.1)	Wu et al., 2011; Buszard et al., 2013

UAS-Ptp61F$^{isoB4.1}$	*Ptp61Fn*$^{4.1}$	Coral Warr	Nuclear-localised isoform, 3rd chromosome insertion (4.1)	Wu et al., 2011; Buszard et al., 2013
equatorial-GAL4	*eq-GAL4*	Henry Sun		Tang and Sun, 2002
10×Stat92E-GFP	*10×Stat-GFP*	Erica Bach		Bach et al. 2007
UAS-myrRFP	*UAS-RFP*	BDSC		
eyFLP ; ; Actin>CD2>GAL4, UAS-GFP	*EAG*, *eyFLPout*, *eyFLP ; Act>>GAL4*	Generated from combining eyFLP with the Act>>GAL4 UAS-GFP (obtained from Laura Johnston)		
eyFLP ; UAS-Ras85D^{V12}*, UAS-dlg1*$^{RNAi\ (v41134)}$ */ CyO, tub-GAL80 ; Actin>CD2>GAL4, UAS-GFP*	*EAGRD*	Konrad Basler		Willecke et al., 2011
UAS-Ptp61F RNAi	*Ptp61F*^{RNAi}	VDRC	v37436	
UAS-Stat92E RNAi		VDRC	v43866	
UAS-Socs44A RNAi		VDRC	v102764	
UAS-Socs36E RNAi		VDRC	v52182	
UAS-Dicer-2	*Dcr2*	VDRC	v60009	
UAS-luciferase RNAi		BDSC	31603	
