# Supplementary material for: PTP61F Mediates Cell Competition and Mitigates Tumorigenesis
Source: Int J Mol Sci. 2021 Nov 25;22(23):12732. doi: 10.3390/ijms222312732 (PMC8657627; doi:10.3390/ijms222312732)
Supplement: Supplementary file 1 [file ijms-22-12732-s001.zip › Supplementary Table S2 - image genotypes v3.pdf]

Supplementary Table S2. Genotypes. This table contains the full genotypes of each sample shown in the figures and discussed in the text.

Figure	Panel(s)	Genotype
1	A,A',C,C'	$hsFLP^{122}$; ; *Ubi-GFP, FRT80B / FRT80B*
	B,B',D,D'	$hsFLP^{122}$; ; *Ubi-GFP, FRT80B /* $Ptp61F^{\Delta}$*, FRT80B*
2	A	y^-, w^-, $eyFLP^2$; $Act>y^+>GAL4$*, UAS-GFP / UAS-myrRFP ; FRT82B, tub-GAL80 / UAS-Dcr2, FRT82B*
	B	y^-, w^-, $eyFLP^2$; $Act>y^+>GAL4$*, UAS-GFP /* $UAS\text{-}Ptp61F^{RNAi\ (v37436)}$ *; FRT82B, tub-GAL80 / UAS-Dcr2, FRT82B*
	C	y^-, w^-, $eyFLP^2$; $Act>y^+>GAL4$*, UAS-GFP / UAS-myrRFP ; FRT82B, tub-GAL80 / UAS-Dcr2, FRT82B,* $scrib^1$
	D	y^-, w^-, $eyFLP^2$; $Act>y^+>GAL4$*, UAS-GFP /* $UAS\text{-}Ptp61F^{RNAi\ (v37436)}$ *; FRT82B, tub-GAL80 / UAS-Dcr2, FRT82B,* $scrib^1$
3	A	y^-, w^-, $eyFLP^2$; $Act>y^+>GAL4$*, UAS-GFP / UAS-myrRFP ; FRT82B, tub-GAL80 / UAS-Dcr2, FRT82B*
	B	y^-, w^-, $eyFLP^2$; $Act>y^+>GAL4$*, UAS-GFP /* $UAS\text{-}Ptp61F^{RNAi\ (v37436)}$ *; FRT82B, tub-GAL80 / UAS-Dcr2, FRT82B*
	C	y^-, w^-, $eyFLP^2$; $Act>y^+>GAL4$*, UAS-GFP /* $UAS\text{-}Stat92E^{RNAi\ (v43866)}$ *; FRT82B, tub-GAL80 / UAS-Dcr2, FRT82B*
	D	y^-, w^-, $eyFLP^2$; $Act>y^+>GAL4$*, UAS-GFP /* $UAS\text{-}Ptp61F^{RNAi\ (v37436)}$, $UAS\text{-}Stat92E^{RNAi\ (v43866)}$ *; FRT82B, tub-GAL80 / UAS-Dcr2, FRT82B*
	E	y^-, w^-, $eyFLP^2$; $Act>y^+>GAL4$*, UAS-GFP /* $UAS\text{-}Ptp61F^{RNAi\ (v37436)}$ *; FRT82B, tub-GAL80 / UAS-Dcr2, FRT82B*
	F	y^-, w^-, $eyFLP^2$; $Act>y^+>GAL4$*, UAS-GFP /* $UAS\text{-}Ptp61F^{RNAi\ (v37436)}$ *; FRT82B, tub-GAL80 / UAS-Dcr2, FRT82B,* $scrib^1$
	G	y^-, w^-, $eyFLP^2$; $Act>y^+>GAL4$*, UAS-GFP /* $UAS\text{-}Stat92E^{RNAi\ (v43866)}$ *; FRT82B, tub-GAL80 / UAS-Dcr2, FRT82B,* $scrib^1$
	H	y^-, w^-, $eyFLP^2$; $Act>y^+>GAL4$*, UAS-GFP /* $UAS\text{-}Ptp61F^{RNAi\ (v37436)}$, $UAS\text{-}Stat92E^{RNAi\ (v43866)}$ *; FRT82B, tub-GAL80 / UAS-Dcr2, FRT82B,* $scrib^1$
4	A,F	y^-, w^-, $eyFLP^2$; $Act> y^+>GAL4$*, UAS-GFP / UAS-myrRFP ; FRT82B, tub-GAL80 / FRT82B*
	B	y^-, w^-, $eyFLP^2$; $Act> y^+>GAL4$*, UAS-GFP /* $UAS\text{-}Ptp61Fn^{2.1}$ *; FRT82B, tub-GAL80 / FRT82B*
	C,H	y^-, w^-, $eyFLP^2$; $Act> y^+>GAL4$*, UAS-GFP / UAS-myrRFP ; FRT82B, tub-GAL80,* $scrib^1$ */ FRT82B*
	D	y^-, w^-, $eyFLP^2$; $Act> y^+>GAL4$*, UAS-GFP /* $UAS\text{-}Ptp61Fn^{2.1}$ *; FRT82B, tub-GAL80,* $scrib^1$ */ FRT82B*

	G	*y^{-}, w^{-}, $eyFLP^{2}$; Act> y^{+}>GAL4, UAS-GFP / UAS-Ptp61F^{RNAi} $^{(v37436)}$; FRT82B, tub-GAL80 / UAS-Dcr2, FRT82B*
	I	*y^{-}, w^{-}, $eyFLP^{2}$; Act> y^{+}>GAL4, UAS-GFP / UAS-Ptp61F^{RNAi} $^{(v37436)}$; FRT82B, tub-GAL80, $scrib^{1}$ / UAS-Dcr2, FRT82B*
5	A	*eyFLP ; UAS-GFP / + ; Actin>CD2>GAL4, UAS-GFP / +*
	B,F	*eyFLP ; UAS-Ras85D^{V12}, UAS-dlg1$^{RNAi\ (v41134)}$ / UAS-GFP ; Actin>CD2>GAL4, UAS-GFP / +*
	C,C’,G	*eyFLP ; UAS-Ras85D^{V12}, UAS-dlg1$^{RNAi\ (v41134)}$ / UAS-Ptp61F^{RNAi} $^{(v37436)}$; Actin>CD2>GAL4, UAS-GFP / +*
	D,D’,H	*eyFLP ; UAS-Ras85D^{V12}, UAS-dlg1$^{RNAi\ (v41134)}$ / UAS-Ptp61Fn$^{2.1}$; Actin>CD2>GAL4, UAS-GFP / +*
6	A,C	*eyFLP ; UAS-Ras85D^{V12}, UAS-dlg1$^{RNAi\ (v41134)}$ / + ; Actin>CD2>GAL4, UAS-GFP / Stat92E-lacZ*
		eyFLP ; UAS-Ras85D^{V12}, UAS-dlg1$^{RNAi\ (v41134)}$ / UAS-Ptp61F^{RNAi} $^{(v37436)}$; Actin>CD2>GAL4, UAS-GFP / Stat92E-lacZ
		eyFLP ; UAS-Ras85D^{V12}, UAS-dlg1$^{RNAi\ (v41134)}$ / UAS-Ptp61Fn$^{2.1}$; Actin>CD2>GAL4, UAS-GFP / Stat92E-lacZ
		eyFLP ; + / +, tub-GAL80 ; Actin>CD2>GAL4, UAS-GFP / Stat92E-lacZ
		eyFLP ; + / UAS-Ptp61F$^{RNAi\ (v37436)}$, tub-GAL80 ; Actin>CD2>GAL4, UAS-GFP / Stat92E-lacZ
		eyFLP ; + / UAS-Ptp61Fn$^{2.1}$; Actin>CD2>GAL4, UAS-GFP / Stat92E-lacZ
7	A,A’,A’’	*10×Stat92E-GFP / + ; eq-GAL4, UAS-RFP / +*
	B,B’,B’’	*10×Stat92E-GFP / UAS-Ras85D^{V12}, UAS-dlg1$^{RNAi\ (v41134)}$; eq-GAL4, UAS-RFP / +*
	C,C’,C’’	*10×Stat92E-GFP / UAS-Ras85D^{V12}, UAS-dlg1$^{RNAi\ (v41134)}$; eq-GAL4, UAS-RFP / UAS-Ptp61Fn$^{4.1}$*
	D,D,’,D’’	*10×Stat92E-GFP / UAS-Ras85D^{V12}, UAS-dlg1$^{RNAi\ (v41134)}$; eq-GAL4, UAS-RFP / Ptp61F$^{\Delta}$*
Supplementary Figure S2	A,F	*y^{-}, w^{-}, $eyFLP^{2}$; Act>y^{+}>GAL4, UAS-GFP / UAS-myrRFP ; FRT82B, tub-GAL80 / UAS-Dcr2, FRT82B*
	B	*y^{-}, w^{-}, $eyFLP^{2}$; Act>y^{+}>GAL4, UAS-GFP / UAS-Socs44A^{RNAi} $^{(v102764)}$; FRT82B, tub-GAL80 / UAS-Dcr2, FRT82B*
	C,H	*y^{-}, w^{-}, $eyFLP^{2}$; Act>y^{+}>GAL4, UAS-GFP / UAS-myrRFP ; FRT82B, tub-GAL80 / UAS-Dcr2, FRT82B, $scrib^{1}$*
	D	*y^{-}, w^{-}, $eyFLP^{2}$; Act>y^{+}>GAL4, UAS-GFP / UAS- Socs44A^{RNAi} $^{(v102764)}$; FRT82B, tub-GAL80 / UAS-Dcr2, FRT82B, $scrib^{1}$*
	G	*y^{-}, w^{-}, $eyFLP^{2}$; Act>y^{+}>GAL4, UAS-GFP / UAS-Socs36E^{RNAi} $^{(v52182)}$; FRT82B, tub-GAL80 / UAS-Dcr2, FRT82B*

	I	*y^-, w^-, eyFLP^2 ; Act>y^+>GAL4, UAS-GFP / UAS- Socs36E$^{RNAi\ (v52182)}$; FRT82B, tub-GAL80 / UAS-Dcr2, FRT82B, scrib^1*
