# Supplementary figures and images for: PTP61F Mediates Cell Competition and Mitigates Tumorigenesis
Source: Int J Mol Sci. 2021 Nov 25;22(23):12732. doi: 10.3390/ijms222312732 (PMC8657627; doi:10.3390/ijms222312732)

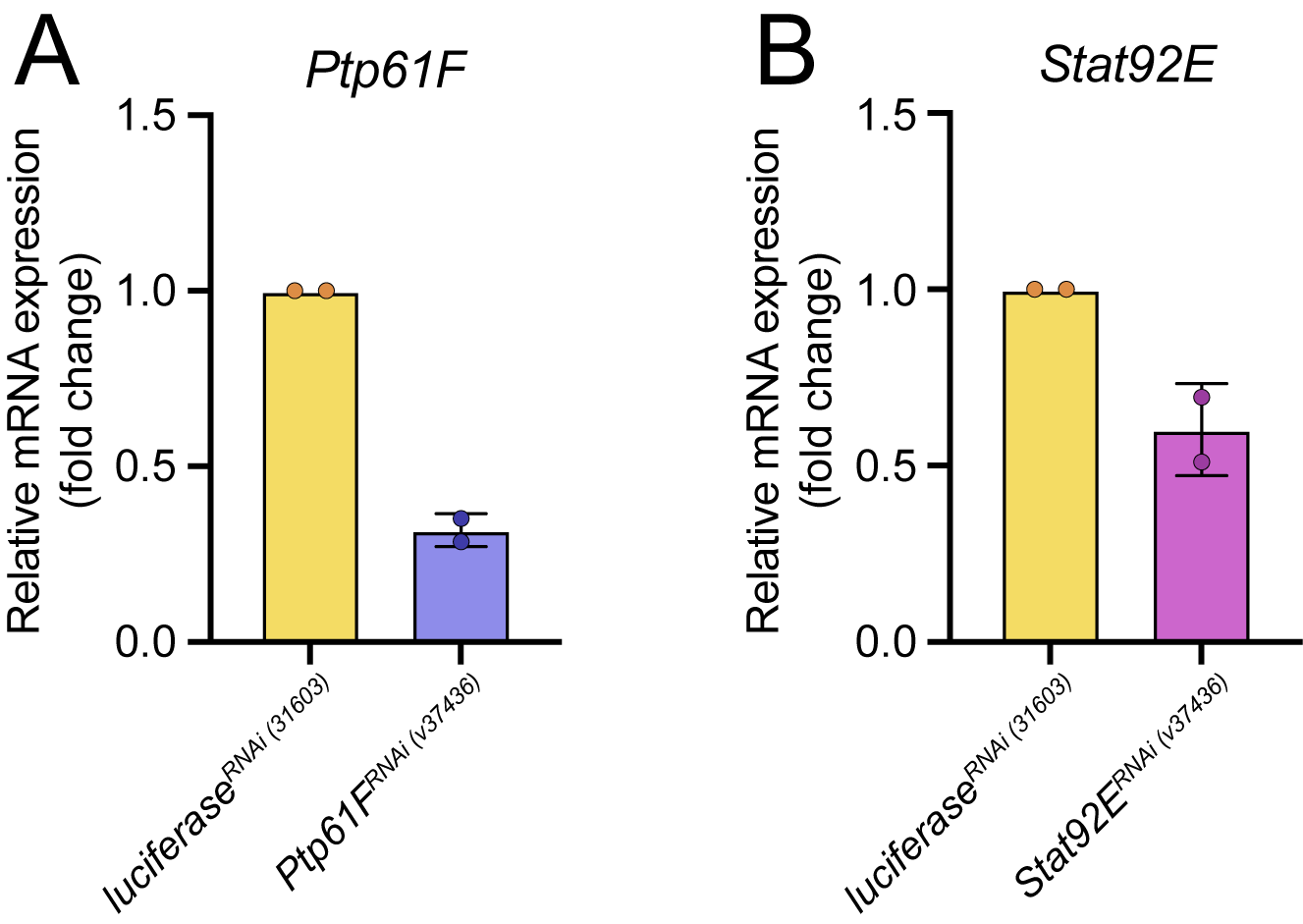

Supplement: Supplementary file 1 [file ijms-22-12732-s001.zip › Supplementary Figure S1 - Ptp61F,Stat92E qRT-PCR.tif]

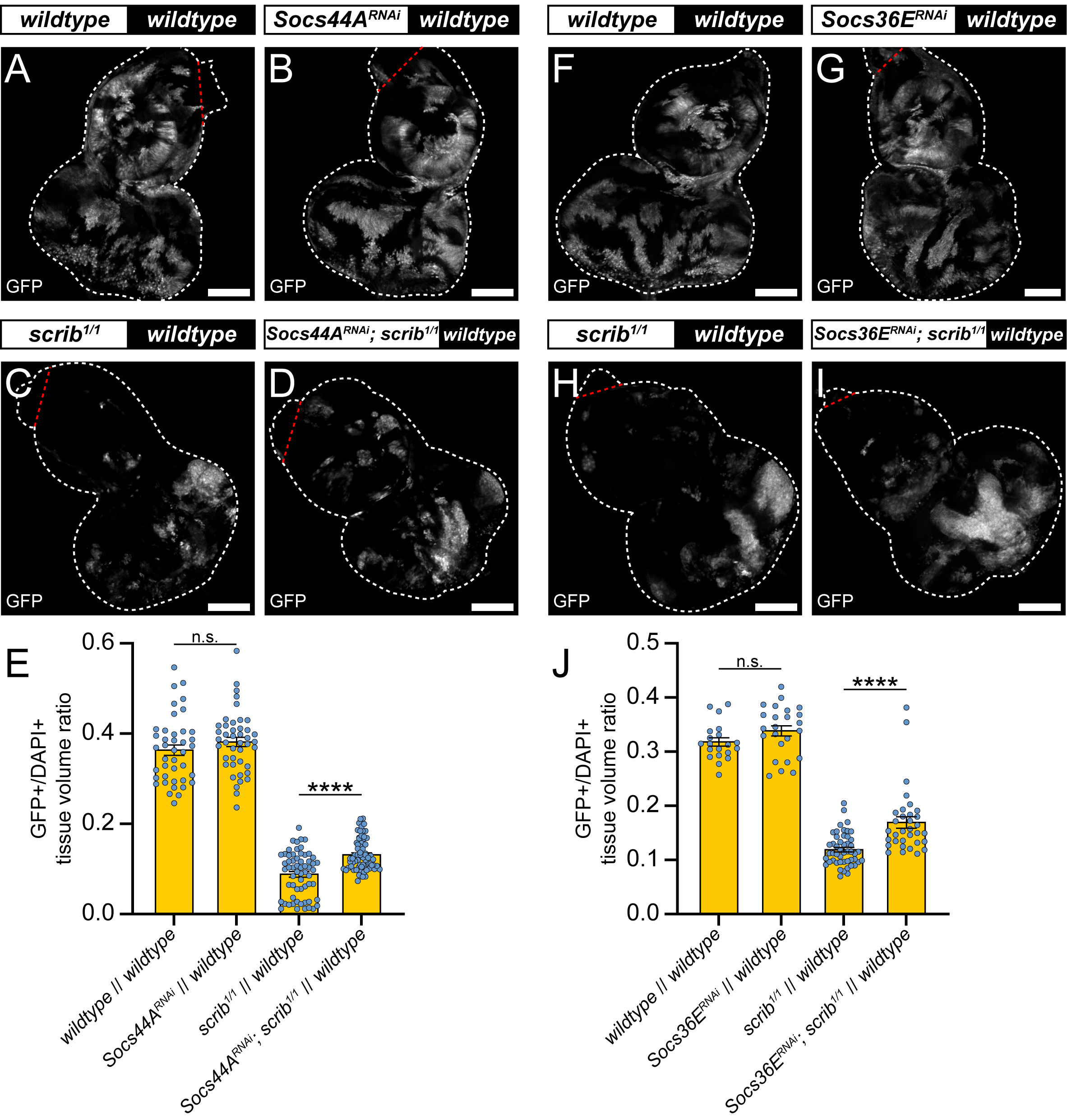

Supplement: Supplementary file 1 [file ijms-22-12732-s001.zip › Supplementary Figure S2 - Socs44A, Socs36E controls.tif]
